# Crystal structure of tetra­kis­(1-oxidopyridin-2-yl)methane methanol tetra­solvate

**DOI:** 10.1107/S2056989015016862

**Published:** 2015-09-12

**Authors:** Kouzou Matsumoto, Kazuaki Kawashita, Masaki Kannami, Masaji Oda

**Affiliations:** a Institute of Natural Sciences, Senshu University, Higashimita 2-1-1, Kawasaki, Kanagawa 214-8580, Japan; b Department of Chemistry, Graduate School of Science, Osaka University, Toyonaka, Osaka 560-0043, Japan

**Keywords:** crystal structure, pyridine *N*-oxide, *S*4 symmetry, hydrogen bonding

## Abstract

The asymmetric unit of the title compound, C_21_H_16_N_4_O_4_·4CH_3_OH, consists of a quarter mol­ecule of tetra­kis­(1-oxidopyridin-2-yl)methane and one methanol solvent mol­ecule. In the crystal, the pyridine *N*-oxide derivative is located about a fourfold rotoinversion axis and exhibits *S*_4_ symmetry along the *c* axis. An inter­molecular O—H⋯O hydrogen bond is observed between the O atom of the pyridine *N*-oxide and the OH group of the methanol. An inter­molecular C—H⋯O bond is also observed between adjacent pyridine *N*-oxide rings.

## Related literature   

For aspects of pyridine *N*-oxides, see: Katritzky & Lagowski (1971 ▸). For reviews of metal complexes of pyridine *N*-oxides, see: Orchin & Schmidt (1968 ▸); Carlin & De Jongh (1986 ▸). For the synthesis of the title compound, see: Matsumoto *et al.* (2003 ▸). For coordination polymers of pyridine *N*-oxides, see: Henkelis *et al.* (2012 ▸). For structures of related mol­ecules, see: Betz *et al.* (2011 ▸); Matsumoto *et al.* (2014 ▸). For the effect of the formation of hydrogen bonds on the N—O bond length of pyridine *N*-oxides, see: Eichhorn (1987 ▸).
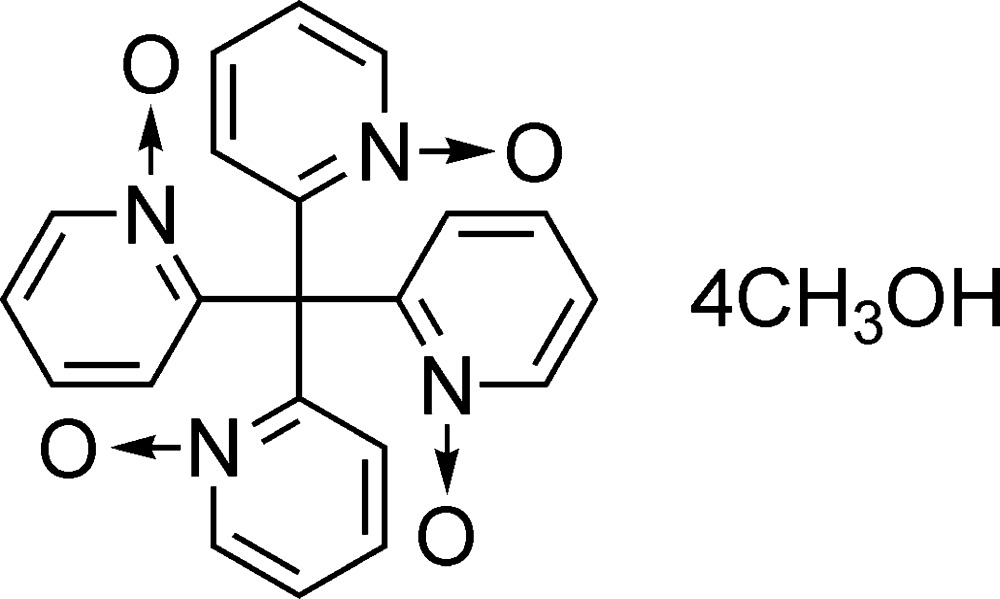


## Experimental   

### Crystal data  

C_21_H_16_N_4_O_4_·4CH_4_O
*M* *_r_* = 516.54
Tetragonal, 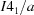

*a* = 14.4474 (4) Å
*c* = 12.2965 (5) Å
*V* = 2566.62 (18) Å^3^
*Z* = 4
Mo *K*α radiation
μ = 0.10 mm^−1^
*T* = 200 K
0.2 × 0.2 × 0.1 mm

### Data collection  

Rigaku R-AXIS RAPID diffractometer
12321 measured reflections
1470 independent reflections
1289 reflections with *I* > 2σ(*I*)
*R*_int_ = 0.031

### Refinement  

*R*[*F* ^2^ > 2σ(*F* ^2^)] = 0.049
*wR*(*F* ^2^) = 0.138
*S* = 1.07
1470 reflections
86 parameters
H-atom parameters constrained
Δρ_max_ = 0.40 e Å^−3^
Δρ_min_ = −0.25 e Å^−3^

### 

Data collection: *PROCESS-AUTO* (Rigaku, 1998 ▸); cell refinement: *PROCESS-AUTO*; data reduction: *PROCESS-AUTO*; program(s) used to solve structure: *SHELXS97* (Sheldrick, 2008 ▸); program(s) used to refine structure: *SHELXL2014* (Sheldrick, 2015 ▸); molecular graphics: *Yadokari-XG 2009* (Wakita, 2001 ▸) and *ORTEP-3 for Windows* (Farrugia, 2012 ▸); software used to prepare material for publication: *Yadokari-XG 2009* and *publCIF* (Westrip, 2010 ▸).

## Supplementary Material

Crystal structure: contains datablock(s) I, Global. DOI: 10.1107/S2056989015016862/is5417sup1.cif

Structure factors: contains datablock(s) I. DOI: 10.1107/S2056989015016862/is5417Isup2.hkl

Supporting information file. DOI: 10.1107/S2056989015016862/is5417Isup3.cml

**Fig. 1.**
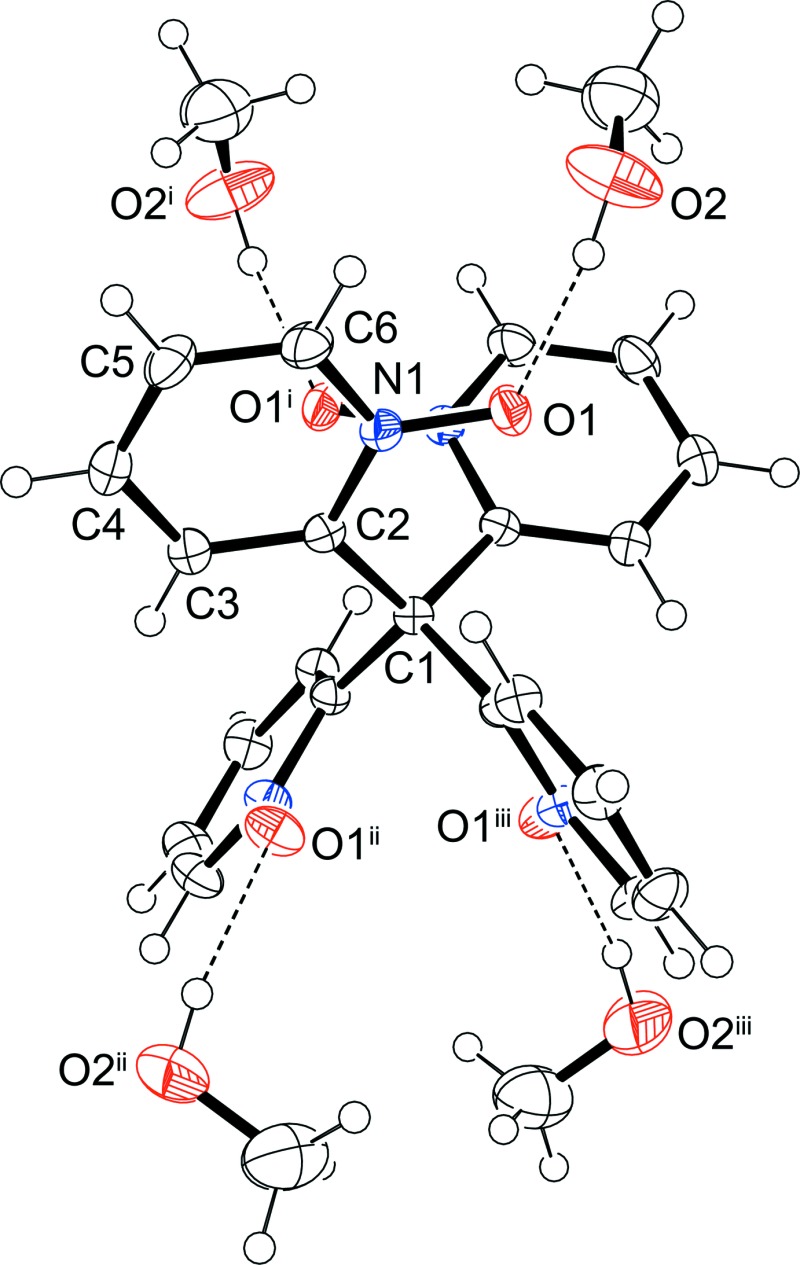
ORTEP drawing of the title compound (viewed along the b axis). Displacement ellipsoids are drawn at the 50% probability level. The hydrogen bonds are shown in the dashed lines. [Symmetry codes: (i) -x, 1/2 - y, z; (ii) -1/4 + y, 1/4 - x, 5/4 - z; (iii) 1/4 - y, 1/4 + x, 5/4 - z.]

Click here for additional data file.ORTEP b x y z y x z y x z . DOI: 10.1107/S2056989015016862/is5417fig1.tif

*ORTEP* drawing of the title compound (viewed along the *b* axis). Displacement ellipsoids are drawn at the 50% probability level. The hydrogen bonds are shown in the dashed lines. [Symmetry codes: (i) −*x*, 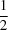 − *y*, *z*; (ii) −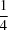 + *y*, 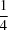 − *x*, 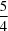 − *z*; (iii) 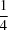 − *y*, 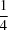 + *x*, 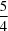 − *z*.]

CCDC reference: 1423138

Additional supporting information:  crystallographic information; 3D view; checkCIF report

## Figures and Tables

**Table 1:** Hydrogen-bond geometry (, )

*D*H*A*	*D*H	H*A*	*D* *A*	*D*H*A*
O2H5O1	0.84	1.90	2.7285(15)	169
C4H2O1^i^	0.95	2.37	3.290(2)	163

Symmetry code: (i) 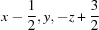.

## supplementary crystallographic information

### S1. Comment

Pyridine *N*-oxides are one of the most common heterocyclic *N*-oxides and their physical and chemical properties are studied in detail (Katritzky & Lagowski, 1971). Pyridine *N*-oxides are the important compounds not only as the precursors of the substituted pyridine derivatives but also as the ligand molecules for the metal complexes (Orchin & Schmidt, 1968; Carlin & De Jongh, 1986). Recently, the bridging ligands containing more than two pyridine *N*-oxide groups were explored (Henkelis *et al.*, 2012). In the course of our investigation of tetrakis(pyridin-2-yl)methane (Matsumoto *et al.*, 2003), we are interested in the corresponding *N*-oxides as the bridging ligand and now we report the crystal structure of the title compound (Fig. 1). The bond lengths and angles of pyridine rings are similar to those of 2-methylpyridine *N*-oxide (Betz *et al.*, 2011). The C1—C2 bond length [1.5472 (11) Å] is similar to that of tetrakis(pyridin-2-yl)methane [1.545 (2) Å] (Matsumoto *et al.*, 2014) and the prominent C1—C2 bond elongation by *N*-oxidation is not observed. The N—O bond length [1.3174 (14) Å] is also normal value in considering the formation of hydrogen bond with methanol molecule (Eichhorn, 1987). The interatomic distance of hydrogen bond is O1···H5 = 1.90 Å [O1···O2 = 2.7285 (15) Å]. An intermolecular C—H···O bond is also observed between the adjacent pyridine *N*-oxide rings [C4···H2 = 2.37 Å, O1···O2 = 3.290 (2) Å; Table 1].

### S2. Experimental

To a solution of tetrakis(pyridin-2-yl)methane (100 mg, 0.3 mmol) in acetic acid (4.5 mL) was added 30% aqueous solution of hydrogen peroxide (66 mmol). The mixture was heated to 90 °C for 2.5 hours. After cooling to room temperature, acetone (20 mL) was added. When the mixture was stirred a few minutes, white precipitates appeared. Collection of the precipitate by filtration gave the title compound (120 mg, 46%) as colourless solid. The single crystals were prepared by slow evaporation of a solution of the title compound in methanol. The obtained single crystals were highly efflorescent and the exposure of the crystal to the air should be avoided.

### S3. Refinement

H atoms were positioned geometrically and allowed to ride on their parent atoms, with C—H = 0.95 Å and *U*_iso_(H) = 1.2*U*_eq_(C) for aromatic H atoms, with C—H = 0.98 Å and *U*_iso_(H) = 1.5*U*_eq_(C) for methyl H atoms, and with O—H = 0.84 Å and *U*_iso_(H) = 1.5*U*_eq_(O) for hydroxyl H atoms.

### Crystal data

**Table:**

C_21_H_16_N_4_O_4_·4CH_4_O	*D*_x_ = 1.337 Mg m^−^^3^
*M**_r_* = 516.54	Mo *K*α radiation, λ = 0.71075 Å
Tetragonal, *I*4_1_/*a*	Cell parameters from 9662 reflections
*a* = 14.4474 (4) Å	θ = 3.6–27.4°
*c* = 12.2965 (5) Å	µ = 0.10 mm^−^^1^
*V* = 2566.62 (18) Å^3^	*T* = 200 K
*Z* = 4	Prism, colourless
*F*(000) = 1096	0.2 × 0.2 × 0.1 mm

### Data collection

**Table:**

Rigaku R-AXIS RAPID diffractometer	*R*_int_ = 0.031
Detector resolution: 10.00 pixels mm^-1^	θ_max_ = 27.4°, θ_min_ = 3.6°
ω scans	*h* = −18→18
12321 measured reflections	*k* = −18→18
1470 independent reflections	*l* = −15→15
1289 reflections with *I* > 2σ(*I*)	

### Refinement

**Table:**

Refinement on *F*^2^	Primary atom site location: structure-invariant direct methods
Least-squares matrix: full	Secondary atom site location: difference Fourier map
*R*[*F*^2^ > 2σ(*F*^2^)] = 0.049	Hydrogen site location: inferred from neighbouring sites
*wR*(*F*^2^) = 0.138	H-atom parameters constrained
*S* = 1.07	*w* = 1/[σ^2^(*F*_o_^2^) + (0.0821*P*)^2^ + 1.7364*P*] where *P* = (*F*_o_^2^ + 2*F*_c_^2^)/3
1470 reflections	(Δ/σ)_max_ < 0.001
86 parameters	Δρ_max_ = 0.40 e Å^−^^3^
0 restraints	Δρ_min_ = −0.25 e Å^−^^3^

### Special details

**Table:**

Geometry. All e.s.d.'s (except the e.s.d. in the dihedral angle between two l.s. planes) are estimated using the full covariance matrix. The cell e.s.d.'s are taken into account individually in the estimation of e.s.d.'s in distances, angles and torsion angles; correlations between e.s.d.'s in cell parameters are only used when they are defined by crystal symmetry. An approximate (isotropic) treatment of cell e.s.d.'s is used for estimating e.s.d.'s involving l.s. planes.
Refinement. Refinement was performed using all reflections. The weighted *R*-factor (*wR*) and goodness of fit (*S*) are based on *F*^2^. *R*-factor (gt) are based on *F*. The threshold expression of *F*^2^ > 2.0 σ(*F*^2^) is used only for calculating *R*-factor (gt).

### Fractional atomic coordinates and isotropic or equivalent isotropic displacement parameters (Å^2^)

**Table:**

	*x*	*y*	*z*	*U*_iso_*/*U*_eq_	
C1	0.0000	0.2500	0.6250	0.0172 (5)	
C2	−0.06345 (8)	0.18960 (8)	0.69738 (9)	0.0181 (3)	
C3	−0.15822 (8)	0.17917 (8)	0.68249 (10)	0.0218 (3)	
H1	−0.1873	0.2091	0.6228	0.026*	
C4	−0.21147 (9)	0.12608 (9)	0.75266 (11)	0.0263 (3)	
H2	−0.2762	0.1198	0.7416	0.032*	
C5	−0.16814 (10)	0.08235 (10)	0.83934 (12)	0.0299 (4)	
H3	−0.2030	0.0458	0.8887	0.036*	
C6	−0.07458 (9)	0.09250 (10)	0.85293 (11)	0.0276 (3)	
H4	−0.0449	0.0623	0.9120	0.033*	
C7	0.14412 (17)	0.16009 (17)	1.05514 (18)	0.0626 (6)	
H6	0.0822	0.1835	1.0727	0.094*	
H7	0.1792	0.2082	1.0169	0.094*	
H8	0.1764	0.1433	1.1224	0.094*	
N1	−0.02328 (7)	0.14510 (7)	0.78324 (8)	0.0211 (3)	
O1	0.06661 (6)	0.15169 (7)	0.79946 (8)	0.0279 (3)	
O2	0.13633 (12)	0.08248 (11)	0.98897 (12)	0.0639 (5)	
H5	0.1193	0.0987	0.9265	0.096*	

### Atomic displacement parameters (Å^2^)

**Table:**

	*U*^11^	*U*^22^	*U*^33^	*U*^12^	*U*^13^	*U*^23^
C1	0.0179 (7)	0.0179 (7)	0.0159 (10)	0.000	0.000	0.000
C2	0.0194 (6)	0.0181 (5)	0.0167 (6)	0.0004 (4)	0.0014 (4)	0.0005 (4)
C3	0.0202 (6)	0.0224 (6)	0.0230 (6)	0.0003 (4)	−0.0006 (4)	−0.0001 (5)
C4	0.0195 (6)	0.0286 (7)	0.0308 (7)	−0.0018 (5)	0.0037 (5)	−0.0003 (5)
C5	0.0275 (7)	0.0321 (7)	0.0300 (7)	−0.0014 (5)	0.0095 (5)	0.0067 (5)
C6	0.0287 (7)	0.0315 (7)	0.0225 (6)	0.0021 (5)	0.0039 (5)	0.0091 (5)
C7	0.0630 (13)	0.0783 (15)	0.0464 (11)	−0.0129 (11)	−0.0033 (9)	−0.0051 (10)
N1	0.0194 (5)	0.0251 (5)	0.0186 (5)	0.0015 (4)	0.0009 (4)	0.0029 (4)
O1	0.0183 (5)	0.0382 (6)	0.0271 (5)	0.0004 (4)	−0.0033 (3)	0.0090 (4)
O2	0.0954 (12)	0.0553 (9)	0.0411 (8)	0.0033 (7)	−0.0273 (7)	0.0119 (6)

### Geometric parameters (Å, º)

**Table:**

C1—C2	1.5472 (11)	C5—C6	1.3698 (19)
C1—C2^i^	1.5472 (11)	C5—H3	0.9500
C1—C2^ii^	1.5472 (11)	C6—N1	1.3643 (16)
C1—C2^iii^	1.5472 (11)	C6—H4	0.9500
C2—N1	1.3656 (16)	C7—O2	1.390 (3)
C2—C3	1.3895 (16)	C7—H6	0.9800
C3—C4	1.3874 (18)	C7—H7	0.9800
C3—H1	0.9500	C7—H8	0.9800
C4—C5	1.3881 (19)	N1—O1	1.3174 (14)
C4—H2	0.9500	O2—H5	0.8400
			
C2—C1—C2^i^	109.77 (9)	C6—C5—H3	120.3
C2—C1—C2^ii^	109.32 (4)	C4—C5—H3	120.3
C2^i^—C1—C2^ii^	109.32 (4)	N1—C6—C5	121.26 (12)
C2—C1—C2^iii^	109.32 (4)	N1—C6—H4	119.4
C2^i^—C1—C2^iii^	109.32 (4)	C5—C6—H4	119.4
C2^ii^—C1—C2^iii^	109.77 (9)	O2—C7—H6	109.5
N1—C2—C3	118.00 (11)	O2—C7—H7	109.5
N1—C2—C1	117.29 (9)	H6—C7—H7	109.5
C3—C2—C1	124.69 (10)	O2—C7—H8	109.5
C4—C3—C2	121.63 (11)	H6—C7—H8	109.5
C4—C3—H1	119.2	H7—C7—H8	109.5
C2—C3—H1	119.2	O1—N1—C6	118.73 (10)
C3—C4—C5	118.62 (12)	O1—N1—C2	120.13 (10)
C3—C4—H2	120.7	C6—N1—C2	121.13 (11)
C5—C4—H2	120.7	C7—O2—H5	109.5
C6—C5—C4	119.34 (12)		
			
C2^i^—C1—C2—N1	−45.51 (8)	C3—C4—C5—C6	−0.2 (2)
C2^ii^—C1—C2—N1	74.40 (6)	C4—C5—C6—N1	0.3 (2)
C2^iii^—C1—C2—N1	−165.42 (10)	C5—C6—N1—O1	−179.29 (12)
C2^i^—C1—C2—C3	133.05 (13)	C5—C6—N1—C2	0.0 (2)
C2^ii^—C1—C2—C3	−107.04 (14)	C3—C2—N1—O1	178.83 (10)
C2^iii^—C1—C2—C3	13.14 (11)	C1—C2—N1—O1	−2.51 (15)
N1—C2—C3—C4	0.59 (18)	C3—C2—N1—C6	−0.48 (18)
C1—C2—C3—C4	−177.96 (10)	C1—C2—N1—C6	178.18 (10)
C2—C3—C4—C5	−0.2 (2)		

Symmetry codes: (i) −*x*, −*y*+1/2, *z*; (ii) −*y*+1/4, *x*+1/4, −*z*+5/4; (iii) *y*−1/4, −*x*+1/4, −*z*+5/4.

### Hydrogen-bond geometry (Å, º)

**Table:**

*D*—H···*A*	*D*—H	H···*A*	*D*···*A*	*D*—H···*A*
O2—H5···O1	0.84	1.90	2.7285 (15)	169
C4—H2···O1^iv^	0.95	2.37	3.290 (2)	163

Symmetry code: (iv) *x*−1/2, *y*, −*z*+3/2.

## References

[bb1] Betz, R., Gerber, T. & Schalekamp, H. (2011). *Z. Kristallogr. New Cryst. Struct.* **226**, 603–604.

[bb2] Carlin, R. L. & De Jongh, L. J. (1986). *Chem. Rev.* **86**, 659–680.

[bb3] Eichhorn, K. (1987). *Acta Cryst.* B**43**, 111–112.

[bb4] Farrugia, L. J. (2012). *J. Appl. Cryst.* **45**, 849–854.

[bb5] Henkelis, J. J., Barnett, S. A., Harding, L. P. & Hardie, M. J. (2012). *Inorg. Chem.* **51**, 10657–10674. 10.1021/ic300940k 23016558

[bb6] Katritzky, A. R. & Lagowski, J. M. (1971). In *Chemistry of the Heterocyclic N-Oxides*. New York: Academic Press.

[bb7] Matsumoto, K., Kannami, M., Fuyuhiro, A. & Oda, M. (2014). *Acta Cryst.* E**70**, o1277–o1278. 10.1107/S1600536814025057 PMC4257413 25553043

[bb8] Matsumoto, K., Kannami, M. & Oda, M. (2003). *Tetrahedron Lett.* **44**, 2861–2864.

[bb9] Orchin, M. & Schmidt, P. J. (1968). *Coord. Chem. Rev.* **3**, 345–373.

[bb10] Rigaku (1998). *PROCESS-AUTO.* Rigaku Corporation, Tokyo, Japan.

[bb11] Sheldrick, G. M. (2008). *Acta Cryst.* A**64**, 112–122. 10.1107/S0108767307043930 18156677

[bb12] Sheldrick, G. M. (2015). *Acta Cryst.* C**71**, 3–8.

[bb13] Wakita, K. (2001). *Yadokari-XG.* http://www.hat.hi-ho.ne.jp/k-wakita/yadokari.

[bb14] Westrip, S. P. (2010). *J. Appl. Cryst.* **43**, 920–925.

