# Supplementary figures and images for: Crystal structure of tetra­kis­(1-oxidopyridin-2-yl)methane methanol tetra­solvate
Source: Acta Crystallogr E Crystallogr Commun. 2015 Sep 12;71(Pt 10):o754–5. doi: 10.1107/S2056989015016862 (PMC4647381; doi:10.1107/S2056989015016862)

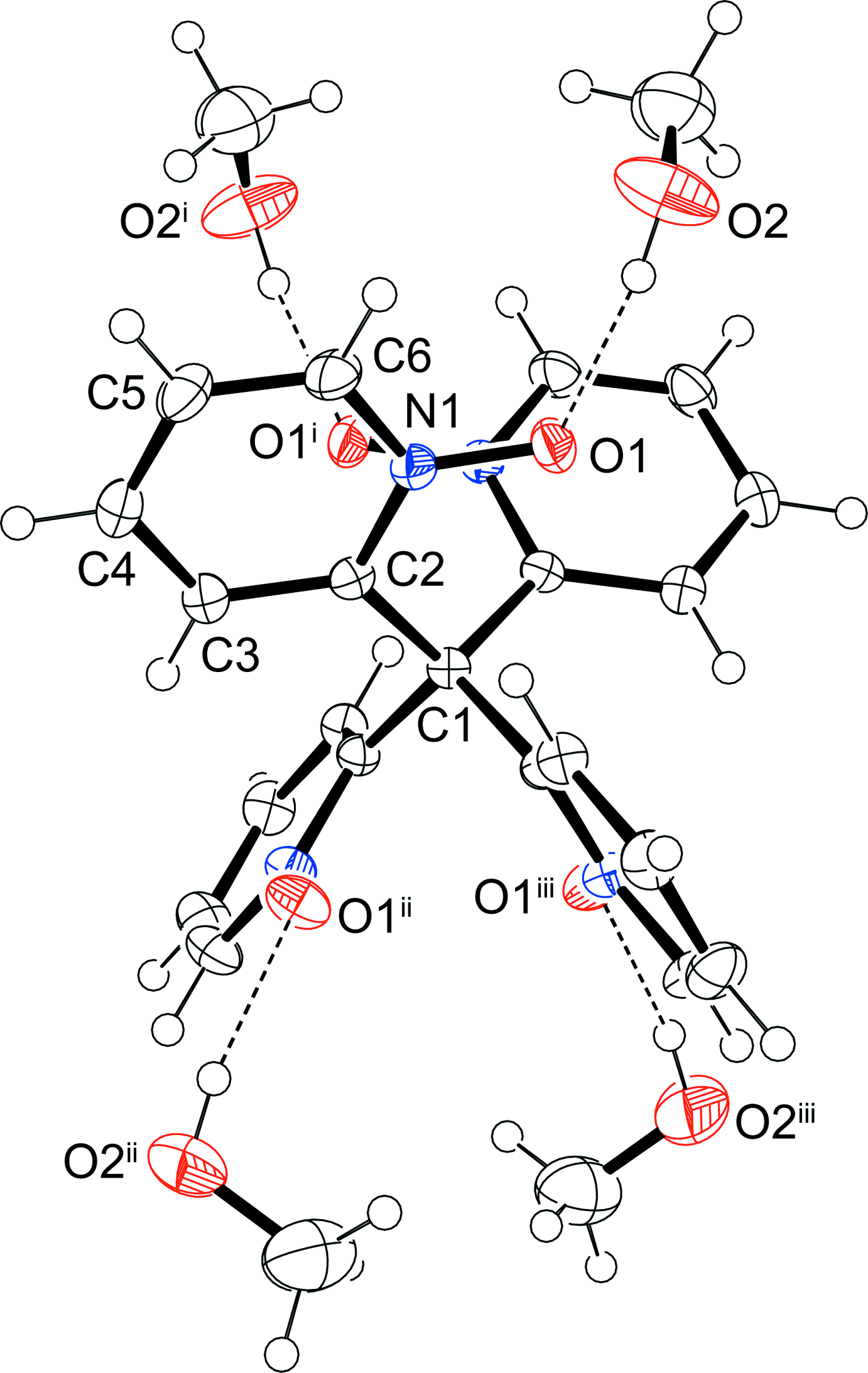

Supplement: Supplementary file 4 [file e-71-0o754-fig1.tif]
